# A Novel Modified Off-Pump Linear Closure Technique of Left Ventricular Aneurysm - A Case Report

**DOI:** 10.21470/1678-9741-2024-0342

**Published:** 2025-09-26

**Authors:** Mustafa Selcuk Atasoy, Ayhan Muduroglu

**Affiliations:** 1 Department of Cardiovascular Surgery, Bursa City Hospital, Bursa, Turkey

**Keywords:** Cardiopulmonary Bypass, Myocardial Infarctation, Angina Pectoris, Heart Failure, Incidence, Aneurysm, Catheters.

## Abstract

Left ventricular aneurysm is an important mechanical complication of myocardial infarction, and its reported incidence after myocardial infarction varies between 10 and 35%. Left ventricular aneurysms in patients with angina pectoris, congestive heart failure, malignant ventricular arrhythmias, and systemic embolization should be surgically repaired. In this paper, we present a novel modified off-pump linear closure technique performed by using a simple Foley catheter for hemostasis on beating heart without cardiopulmonary bypass for the surgical treatment of left ventricular aneurysm. To the best of our knowledge, this is the first reported case of such an approach in the literature.

## INTRODUCTION

**Table:** 

Abbreviations, Acronyms & Symbols	
ACT	= Activated clotting time
CA	= Coronary angiography
CABG	= Coronary artery bypass grafting
CAD	= Coronary artery disease
CPB	= Cardiopulmonary bypass
Cx	= Circumflex
EVPP	= Endoventricular patch plasty
LAD	= Left anterior descending
LVA	= Left ventricular aneurysm
LVEF	= Left ventricular ejection fraction
MI	= Myocardial infarction
RCA	= Right coronary artery
TTE	= Transthoracic echocardiography

Left ventricular aneurysm (LVA) is a distinct area of abnormal left ventricular diastolic contour with systolic dyskinesia or paradoxical bulging which reduces left ventricular ejection fraction (LVEF). The reported incidence of LVA following myocardial infarction (MI) varies between 10 and 35%, and its absolute incidence has recently decreased on account of the early treatment of MI with thrombolytics and revascularization. The surgical treatment should be performed in the existence of angina pectoris, congestive heart failure, malignant ventricular arrhythmias, and systemic embolization^[1,2]^. There are various types of repair techniques such as plication, linear closure, circular patch, and endoventricular patch, which have been performed in the surgical treatment of LVA^[1-3]^. In this paper, a novel modified linear closure technique performed by using a simple Foley catheter to provide hemostasis on beating heart without use of cardiopulmonary bypass (CPB) for the surgical treatment of LVA is presented.

## CASE PRESENTATION

A 64-year-old male patient was referred to our outpatient clinic with complaints of exertional chest pain and dyspnea. His medical history revealed the diagnosis of hypertension and diabetes mellitus as well as an attack like MI three years before. He received transthoracic echocardiography (TTE) and coronary angiography (CA). TTE revealed LVEF of 0.50, mild mitral regurgitation, LVA measuring 3 × 4.5 cm on inferior wall, and appearance of doubtful existence of thrombus in the aneurysm sac. CA revealed three-vessel coronary artery disease (CAD) with a 100% stenosis in the distal right coronary artery (RCA), an 80-90% stenosis in the proximal left anterior descending (LAD) artery, and a 70% stenosis in the proximal circumflex (Cx) artery. It was decided to perform a coronary artery bypass grafting (CABG) operation as well as probably a surgical repair of LVA, and he was transferred to the operating room after informing him about the operation and obtaining the surgical consent.

### Surgical Technique

**Fig. 1:**
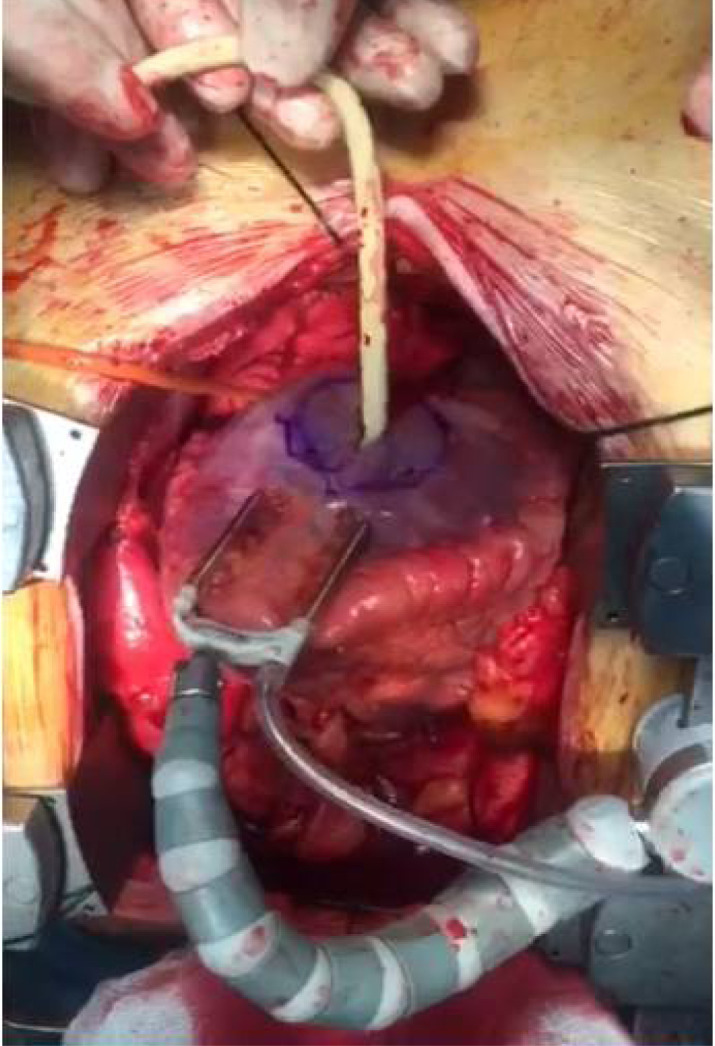
View of aneurysm before repair when the Foley catheter was placed in the neck of aneurysm.

**Fig. 2A and 2B:**
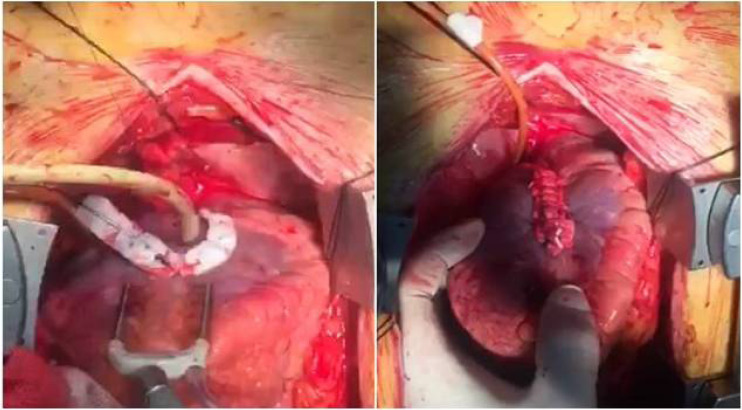
View of aneurysm at the time of surgical repair and after repair is completed.

The patient was operated on in the supine position under general anesthesia. After median sternotomy and pericardiotomy, the heart was explored and thin-walled and dyskinetic LVA formation on inferior wall was observed. Firstly, on-pump surgery with CPB was planned to perform for the procedure; however, after heparin administration of 350 IU/kg, the target activated clotting time (ACT) level could not be reached, then despite additional repetitive heparin bolus as well as a total of 3 U fresh frozen plasma administration, the maximum ACT level was measured as 287 seconds, and current clinical condition is considered as significant heparin resistance. Therefore, the surgical procedure compulsorily continued without CPB. Off-pump CABG × 3 (left internal thoracic artery-LAD, aorto-Cx obtuse branch, aorto-RCA posterior descending branch) procedure was performed first. Afterwards, on beating heart without CPB, left ventricular apex was hold up and stabilized by using Octopus™ IV tissue stabilizer (Maquet®) to facilitate the visualization of aneurysm, and the borders of aneurysm were drawn by a pen. Shortly after, an approximately 1 cm small incision was made in the middle of the aneurysm sac, and digital palpation was carried out in order to control the initial low-flow hemorrhage. No thrombus was roughly seen beyond the incision at first sight. A Foley catheter was placed in the aneurysm through this small incision, and then the balloon at the tip of Foley catheter was inflated to cover the neck of aneurysm for the purpose of hemostasis (Figure 1) (Video 1). The small incision was longitudinally extended not to exceed the borders of aneurysm. Aneurysmal cavity was controlled again in terms of the existence of thrombus; it was confirmed that there were no thrombus materials in the aneurysmal cavity in completely hemostatic field during the exploration. The aneurysm was then closed in a longitudinal line between two layers of Teflon® felt with mattress sutures. An additional over-and-over suture was placed over the felt strips to reinforce the suture line (linear closure technique) (Figures 2A and 2B) (Videos 2, 3, 4, 5, and 6). The manipulations that may increase mitral regurgitation were avoided as much as possible during the aneurysm repair. Following the aneurysm repair, an intraoperative transesophageal echocardiographic examination was obtained, and it revealed that mild mitral regurgitation continued identically and did not progress. Afterwards, the operation was completed in a standard fashion. During the operation, close hemodynamic follow was carried out in coordination with the anesthesia team. The patient was hemodynamically stable from the beginning to the end of the surgery (systolic blood pressure was between 90 and 110 mmHg, diastolic blood pressure was between 50 and 65 mmHg, heart rate was between 70 and 90/min, and oxygen saturation was between 96 and 100%), and the heart did not fail or fibrillate during all operation. Concerning stabilization of the heart, Foley catheter balloon filling into the ventricle below the neck of the aneurysm or other factors did not affect the hemodynamic parameters. After the operation, the patient was transferred to an intensive care unit and monitored closely. During the postoperative period, general and hemodynamic status of the patient was stable, no adverse event occurred, and he was discharged at postoperative fifth day. There were the same LVEF of 0.50 and no mitral regurgitation in the echocardiography control performed two years after the operation.

**Video 1:**
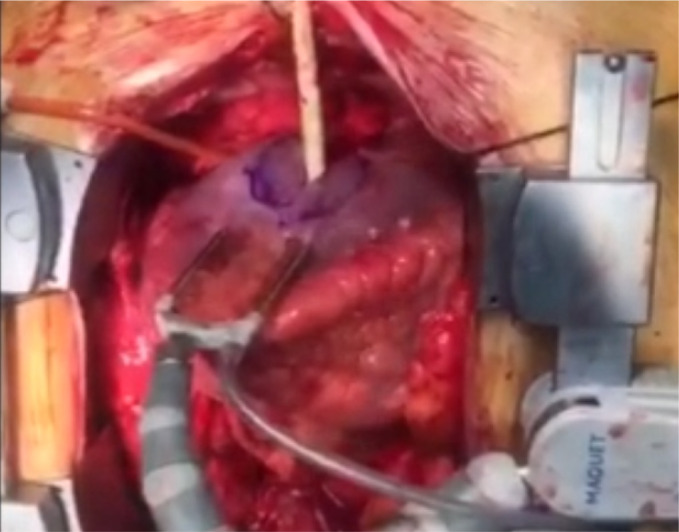
Aneurysm before repair when the Foley catheter was placed in the neck of aneurysm.

*Link: https://youtu.be/_xzd1NO2y6o*

**Video 2:**
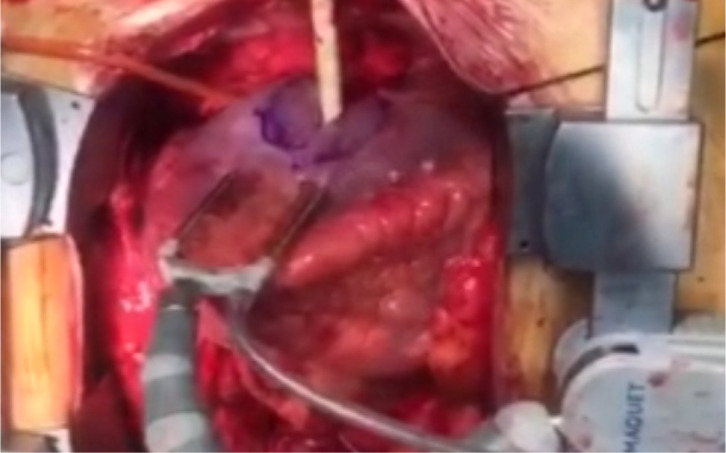
Aneurysm at the time of surgical repair.

*Link: https://youtu.be/crbKJ40evyY*

**Video 3:**
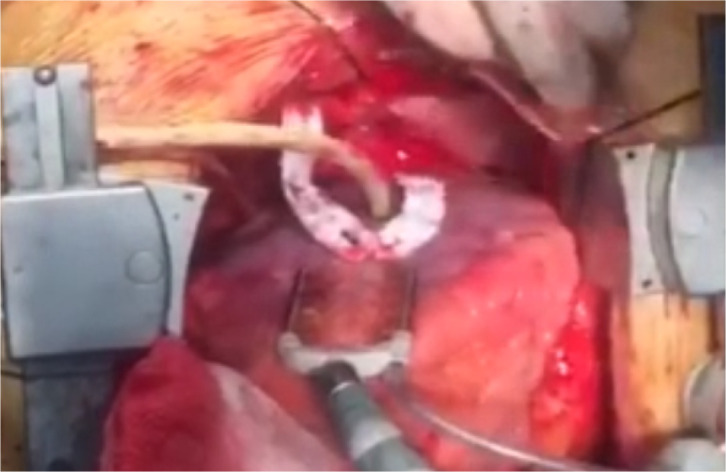
Aneurysm at the time of surgical repair.

*Link: https://youtu.be/syrTi4uPdBQ*

**Video 4:**
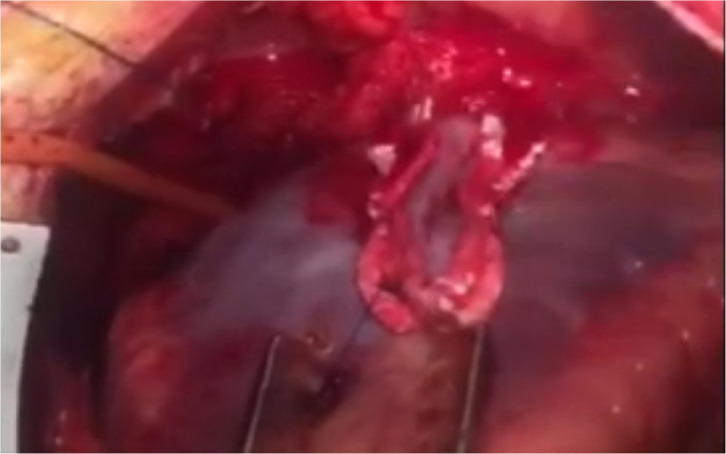
Aneurysm at the time of surgical repair.

*Link: https://youtu.be/tbkDpYlM2UY*

**Video 5:**
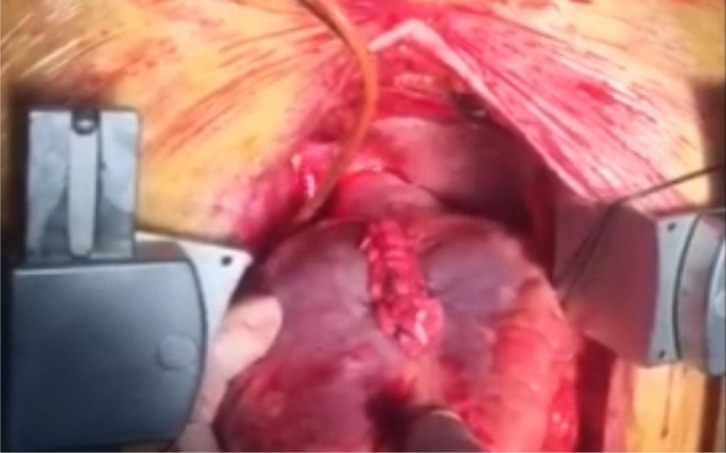
Aneurysm after repair is completed.

*Link: https://youtu.be/1zF7FTX9Sug*

**Video 6:**
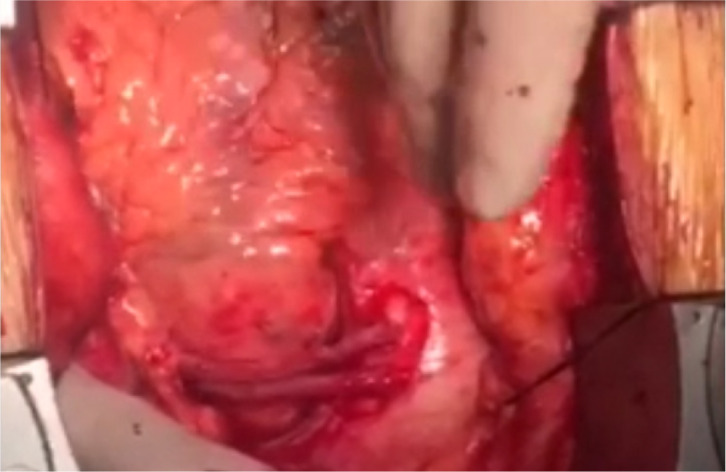
Aneurysm after repair is completed.

*Link: https://youtu.be/pxHuEkuE60k*

## DISCUSSION

LVA is an important mechanical complication which can be observed following MI. Although the precise mechanism of its occurrence is not yet well known, it is known that transmural infarction plays a key role in its pathogenesis. The infarcted myocardium is converted to thin localized scar tissue which exhibits akinetic or paradoxical motion during left ventricular systole^[2,4,5]^. Surgical treatment of LVA is generally not indicated for asymptomatic patients on account of its benign nature. In order to alleviate symptoms and prolong survival, surgery is indicated when at least one of the following four clinical conditions exists: angina pectoris, congestive heart failure, malignant ventricular arrhythmias, or systemic embolization^[1-3]^. In the present case, the primary surgical indication was not the existence of LVA, and we primarily operated the patient for performing CABG procedure; nevertheless, due to the existence of a thin-walled and dyskinetic aneurysm, we also performed concomitant LVA repair.

Surgical repair for LVA has been performed for more than half a century. The main purposes of surgical repair are to correct the size and geometry of the left ventricle, reduce wall tension and paradoxical movement, restore left ventricular function, and improve systolic function^[1,3]^. The first successful LVA excision was performed by Likoff and Bailey by using a special clamp without CPB in 1955^[6]^. In 1958, the first aneurysmectomy with linear repair was successfully performed using CPB by Cooley et al.^[7]^. Ever since, this operation has been widely performed and remained as standard procedure for the surgical treatment of LVA until the late 1980s. In this process, many modified surgical techniques have been devised by creative surgeons. The endoventricular patch plasty (EVPP) technique was introduced as a more physiologic repair than the standard linear repair technique especially when LVA enlarges into the septum^[2,8]^. Nevertheless, there is still a controversy concerning the optimal surgical technique for LVA repair nowadays. While some studies have advocated that EVPP technique is a better surgical choice than linear repair^[2,9,10]^, others have suggested that both techniques have comparable survival rates and postoperative results^[11-13]^. As a result, according to the literature, the superiority of EVPP technique *vs.* standard linear repair has not been exactly proven. In our case, we performed an off-pump CABG for the surgical treatment of three-vessel CAD, and an additional non-standard linear repair for LVA in which a simple Foley catheter was used in order to provide hemostasis on beating heart. Our goal was initially to check the existence of thrombus via a small incision in this case. The first exploration via this small incision already revealed the absence of thrombus roughly. Via this approximately 1 cm incision, we performed initial ventriculotomy that could easily be controlled by applying digital palpation (and probably subsequent purse-string suturing). Afterwards, with an instant thought we intended to provide short-term hemostasis with a Foley catheter and placed the Foley catheter into the aneurysm. The connection between the aneurysm sac and left ventricle was then interrupted when the inflated balloon at the tip of Foley catheter completely covered the neck of aneurysm. We took advantage of the cessation of blood flow into the aneurysm sac as a result of the inflation of balloon at the tip of Foley catheter in the narrow neck. We then extended the ventriculotomy incision and confirmed the absence of thrombus. Afterwards, linear repair of LVA was easily performed on beating heart without CPB. In addition to that the definite superiority of EVPP technique has not yet been proven, in the present operation we also aimed shorter operation time to avoid additional potential risks of prolonged off-pump surgery; therefore, we preferred the linear repair technique instead of EVPP and performed a shorter and safe operation. Moreover, there was no increased mitral insufficiency in control echocardiographic examinations. On the other hand, we preferred open plication technique instead of external plication because of the doubtful thrombus existence.

In fact, we took a serious risk because of thrombus suspicion in the present case, therefore we could be criticized; but we ultimately continued the procedure. As a consequence, although the described procedure carried a serious risk, we wanted to emphasize the feasibility of such surgical approach in the ventricular aneurysm cases without intra-aneurysmal thrombus. On the other hand, this novel technique provides a significant decrease in the detrimental effects of CPB on cardiovascular, pulmonary, neurological, renal, and hematological systems, and also a cost decrease. To the best of our knowledge, this is the first reported case of such an approach in the literature.

## CONCLUSION

Although we do not claim to be routinely applied in this manner, the described modified linear repair technique performed on beating heart without CPB can be successfully performed as an alternative to standard linear closure technique in some unexpected and tough situations as well as in case of small ventricular aneurysms with narrow neck without intra-aneurysmal thrombus. Moreover, certain ventricular aneurysms in selected cases can also be repaired without CPB by using a more functional covering system that will be developed instead of the Foley catheter that we used.
